# Workplace bullying and general health status among the nursing staff of Greek public hospitals

**DOI:** 10.1186/s12991-016-0097-z

**Published:** 2016-03-03

**Authors:** Christina Karatza, Sofia Zyga, Styliani Tziaferi, Panagiotis Prezerakos

**Affiliations:** Faculty of Nursing, University of Peloponnese, E and S Valioti and Plateon, 23100 Sparta, Greece

**Keywords:** General health, Human resources management, Nursing staff, Workplace bullying

## Abstract

**Background:**

The arduous emotional and physical nurses’ work, the gradual nursing staff cutbacks and the lack of recognition that nurses feel regarding their skills and overall capabilities are some of the factors that act of bullying between nursing staff and management, between nurses and patients/families or even among nurses themselves. Workplace bullying has physical and psychological effects on worker-victims and, by extension, patients themselves. The purpose of this study was to investigate the relationship between the phenomenon of workplace bullying and general health status among the nursing staff of Greek public hospitals.

**Methods:**

A cross-sectional study was conducted on a convenience sample of 841 members of the nursing staff working in five major hospitals of the 1st Regional Health Authority of Attica, located in Athens. The response rate was 84.1 %. The respondents completed the Negative Acts Questionnaire (NAQ) and the General Health Questionnaire (GHQ-12) and also their demographic characteristics. The appropriate permissions were obtained by the Hospitals’ Ethics Committees and the questionnaire’s authors. Data were collected from March to July 2013. Data analysis was performed with IBM SPSS 21.0 and included *t* test, *χ*^*2*^ test and regression analysis. The two-tailed significance level was set ≤0.05.

**Results:**

30.2 % of the respondents reported that they had been psychologically harassed in their workplaces during the preceding 6 months. Statistical analysis revealed that relative to other respondents, respondents who had received support from their families and friends enjoyed better health but respondents who perceived their work environments more negatively because of work-related bullying suffered from worse general health.

**Conclusions:**

Workplace bullying among nursing staff is a major concern in Greece. Support systems play a crucial role in addressing the negative effects of bullying and they should be taken into account when designing prevention and troubleshooting policies about bullying.

## Background

The nursing body is the fundamental patient-oriented, care-giving entity; because nurses perform arduous emotional and physical work, frequently under adverse conditions due to resource shortages, they experience work-related burnout to a great extent [1]. Stress symptoms and a hectic pace in the workplace reduce not only the quality of nursing services provided but also nurses’ job satisfaction levels and interest in their work [2, 3]. As a result, nurses develop defence mechanisms that unwittingly lead to forming impersonal relationships with patients; these relationships can quickly degenerate to become openly negative, potentially leading to a variety of deleterious consequences [4]. All of the aforementioned factors, in combination with gradual staff cutbacks and the lack of recognition that nurses feel regarding their skills and overall capabilities, can result in not only work-related burnout syndrome but also acts of bullying between nursing staff and management, between nurses and patients/families or even among nurses themselves [5].

Long-standing conflicts over authority, which are observed within organisations, arise from clashes between values held by the currently extant organisation and styles of management. These conflicts can lead to work-related bullying [6]. Nurses feel a lack of support from the management of their organisations; this feeling is closely related to the occurrence of work-related bullying [7].

Bullying may create and maintain a toxic work environment [8] characterised by decreased work motivation, a lack of concentration, errors and absenteeism; such an environment can result in poor productivity and a low quality of patient care [9–11]. A large number of bullied victims in the nursing profession take a great deal of leave each year in an effort to at least temporarily escape their experiences; this phenomenon results in organisations suffering from reduced profits because the demands of nursing care do not afford these organisations the luxury of losing nurses [12–14]. Bullying destroys victims’ self-confidence and self-image, often driving victims to resign from their positions [15].

Finally, bullying poses a risk to patient safety because it negatively impacts nurses’ main task of providing patient care and disrupts teamwork and communication [16, 17].

Among EU countries, Greece is tied with Spain for the fifth highest rates of workplace harassment, with the highest harassment rates found in Austria and Italy [18]. These data reflect the severity of the situation in our country; however, the Greek Ministry of Health has not implemented appropriate institutional actions to combat the phenomenon of workplace bullying. For all the above-mentioned reasons, this study aimed to explore the relationship between the phenomenon of workplace bullying and the state of general health among the nursing staff of Greek public hospitals and to detect how this relationship differs for nurses with different demographic profiles.

## Methods

### Design

This investigation was a quantitative, cross-sectional study. After a literature review, two self-administered questionnaires were used to achieve the study objectives with the goal of (a) measuring the effects of workplace bullying and (b) measuring the status of general health of the surveyed nursing staff.

### Participants

The final surveyed population, which a convenience sample, consisted of 841 members of the nursing staff working in five major Greek NHS hospitals at the 1st Regional Health Authority of Attica, located in Athens. Data were collected from March 2013 to July 2013. Questionnaires were accompanied by a description of the study’s aims, and clarification was provided when necessary. Completed questionnaires were collected weekly in sealed envelopes. Of the 1000 questionnaires, distributed by the researchers, 841 fully completed questionnaires were returned with an overall response rate of 84.1 %.

### Data collection

After the required permissions were obtained, the following tools were used for the purposes of the study: a. The Negative Acts Questionnaire (NAQ) [19] is one of the most popular tools for measuring perceived exposure to workplace bullying and consists of 23 items organised into the following three sub-scales: (a) personal bullying, (b) work-related bullying and (c) physical bullying. Questionnaire responses were rated on a five-point Likert-type scale, with higher scores representing more negative behaviour. The 23rd item on the questionnaire (“Have you been bullied at work?”), which is not included on any of the three sub-scales, had the following possible answers: (a) no; (b) yes, but only rarely; (c) yes, at times; (d) yes, a few times a week and (e) yes, almost daily. b. The General Health Questionnaire-12 (GHQ-12), which was created in 1970 by Goldberg [20], is primarily used to assess mental health [21, 22]. This tool is simple to use, can be easily completed and rated and has been widely and successfully utilised in myriad mental health studies [23–25]. The GHQ-12 is a multidimensional, simple, quick and reliable tool that focuses on respondents’ inability to cope with everyday activities or with new, painful emotional experiences. The respondent completes the questionnaire to reflect his perceived mental state during the prior several weeks (typically the preceding month). Each item is rated on a four-point scale. The overall questionnaire score is derived from the aggregated scores for each question; higher scores represent a higher degree of psychological distress.

Both questionnaires were accompanied by a brief description of the survey’s objectives and were administered by the first author to the head nurses of each department. A total of 1000 questionnaires in envelopes were distributed, and the response rate was 84.1 %. Survey data were collected over the five-month period from March 2013 to July 2013.

### Ethical considerations

The required approvals were obtained from the relevant competent bodies regarding ethics and from the administration of the 1st Regional Health Authority, which governs the hospitals involved in this study. Participation in the survey was voluntary and respondents’ answers were kept confidential.

### Data analysis

Categorical variables are expressed in terms of absolute (*n*) and relative (%) frequencies, and quantitative variables are expressed in terms of mean, standard deviation, median, minimum and maximum. The Kolmogorov–Smirnov test and normal plots were utilised to test the normality of quantitative variable distribution. All quantitative variables were found to be normally distributed.

Student’s *t* test was used to detect potential relationships between quantitative and dichotomous variables, and analysis of variance was used to detect possible relationships between quantitative variables and categorical variables with >2 categories. Pearson’s correlation coefficient was used to detect potential relationships between two normally distributed quantitative variables.

The Chi-squared (*χ2*) test was used to detect potential relationships between two categorical variables. The Chi-squared (*χ2*) test for trend was used to detect potential relationships between categorical and ordinal variables.

If the dependent variable was quantitative and >2 independent variables were significant at the 0.2 (*p* < 0.2) level in bivariate analysis, multivariate linear regression was applied, using the backward stepwise linear regression model. For multivariate linear regressions, coefficients’ beta values, 95 % confidence intervals and p values are presented.

If the dependent variable was dichotomous and >2 independent variables were significant at the 0.2 (*p* < 0.2) level in bivariate analysis, multivariate logistic regression was applied, using the backward stepwise logistic regression model. For multivariate logistic regressions, odds ratios, 95 % confidence intervals and p values are presented.

Specific post and department were not used as independent variables because of the wide range of possible answers to questions regarding these issues and the exceptionally small number of comments provided in several responses.

Years of prior experience at any hospital and years of prior experience at the hospital, where the survey was conducted, exhibited a high degree of correlation (*r* = 0.9, *p* < 0.001); as a result, years of prior hospital experience were regarded as an independent variable.

A two-tailed significance level of 0.05 was established. Data were analysed using IBM SPSS 21.0 (Statistical Package for the Social Sciences).

### Validity and reliability

A double back-translation process was used to translate both questionnaires into Greek. A pilot study was conducted among 50 nursing personnel to measure each questionnaire’s reliability and face validity. Reliability was measured using Cronbach’s alpha, which was 0.81 and 0.89 for the NAQ and the GHQ-12, respectively. Cronbach’s alpha coefficient is used to assess the reliability (internal consistency) of a scale. Acceptable values of Cronbach’s alpha are higher than 0.7 [26].

These results suggested that the questionnaires exhibited excellent internal consistency. To assess face validity, a convenience sample of health professionals (n = 22) was interviewed regarding the clarity of the questionnaires and any difficulties associated with completing these instruments. The few comments, that were provided, were addressed in the final versions of the questionnaires.

In the main study, Cronbach’s alpha for the NAQ was calculated to be 0.92, suggesting that this questionnaire exhibited excellent internal consistency. Similarly, Cronbach’s alpha for the General Health Questionnaire was found to be 0.90.

## Results and discussion

### Results

Participants’ average age was 40.1 years; 84.7 % of respondents were women, 53.7 % of respondents were married, 56.7 % of respondents had children, 51 % of respondents were higher technological institute graduates and respondents had an average of 16.1 years of prior hospital experience. Overall, 14 % of participants were working in an intensive care unit, 12.5 % of participants were working in a general hospital ward, 9.9 % of participants were working in a casualty department, 8.6 % of participants were working in a respiratory specialty ward, 6.3 % of participants were working in a general surgery ward, 5.8 % of participants were working in nursing administration and 5.7 % of participants were working in a specific surgical practice. Finally, 86.8 % of respondents reported that they were adequately supported by their family environments and 84.8 % of respondents were adequately supported by their friends.

### Negative Acts Questionnaire-12 results

A total of 30.2 % of participants reported that they had been psychologically harassed in their workplaces at various frequencies during the preceding several months (rarely: 17.2 %; occasionally: 9.9 %; a few times per week: 2 %; almost daily: 1.1 %).

The scores regarding the three sub-scales (22 items) of the Negative Acts Questionnaire are shown in Table 1.

**Table 1:** The scores of the three sub-scales of the Negative Acts Questionnaire

Sub-scale	Mean value	Standard deviation	Median value	Min–max value
Personal bullying	19.3	6.8	17	12–56
Work-related bullying	14.2	5.5	13	7–35
Physical bullying	4.4	1.7	4	3–15

Concerning the 23rd item on the questionnaire (“Have you been bullied at work?”), 69.8 % of the participants stated that they have not been bullied at work the past 6 months, 17.2 % stated they have been but only rarely, 9.9 % stated they have been bullied at times, 2.0 % stated they have been bullied a few times a week and the last 1.1 % stated they have been bullied almost daily. Statistically significant relations at a level of 0.20 (*p* < 0.20) were found between marital status, the existence of children, educational level, years of previous hospital employment and support from the familial and friendly environment and psychological harassment in the workplace in the past 6 months.

During the process of conducting bivariate analyses, various statistically significant correlations were found between each NAQ sub-scale and individual features such as marital status, the existence of children, educational standard, years of previous hospital employment and support from the familial and friendly environments. Table 2 provides a synopsis of multivariate linear and logistic regressions between individual features (the independent variables) and NAQ sub-scales (the dependent variables).

**Table 2:** Statistically significant correlations between individual features (independent variables) and the NAQ sub-scales (dependent variables)

Dependent variable	Independent variable	Odds ratio	95 % confidence interval for the odds ratio	*p* value
Physical harassment in the workplace in the past 6 months	The existence of children as opposed to the non-existence of children	1.69	1.26–2.28	0.001
	No support from a familial environment as opposed to the presence of such support	2.06	1.36–3.11	0.001
Personal bullying	The non-existence of children as opposed to the existence of children	1.89	0.98–2.81	<0.001
	No support from a friendly environment as opposed to the existence of such support	2.06	0.80–3.32	0.001
Work-related bullying	The non-existence of children as opposed to the existence of children	1.16	0.41–1.90	0.002
	No support from a familial environment as opposed to the existence of such support	2.04	0.95–3.13	<0.001
Physical bullying	No correlation			

### General health questionnaire results

The average general health score was 12.5 (SD = 6.4), with minimum and maximum values of 0 and 36, respectively. In bivariate analyses, relationships significant at the 0.20 level (*p* < 0.20) were found among gender, support from family and friends and general health scores. Pearson’s correlation coefficients between general health score and personal bullying score, work-related bullying score and physical bullying score were 0.39, 0.47 and 0.29, respectively (*p* < 0.001 in all cases).

In bivariate analyses, relationships significant at the 0.20 level (*p* < 0.20) were found among gender, support from family and friends, scores on the personal bullying, work-related bullying and physical bullying sub-scales and general health scores. For this reason, a multivariate linear regression was performed. The findings from this regression are presented in Table 3.

**Table 3:** Multivariate linear regressions with general health score as an independent variable

	b coefficient	95 % confidence interval for the b coefficient	*p* value
No support from familial environment as opposed to the existence of such support	2.64	1.36–3.92	<0.001
No support from a friendly environment as opposed to the existence of such support	1.76	0.56–2.95	<0.001
Work-related bullying score	0.51	0.44–0.58	0.004

The multivariate logistic regression revealed the following results: a. Respondents who reported that they did not receive support from their familial environments had higher general health scores than respondents who received such support. Thus, participants who reported receiving support from their familial environments enjoyed better overall health than other participants. b. respondents who reported that they did not receive support from their friends had higher general health scores than respondents who received support from friends. c. Higher scores on the work-related bullying sub-scale were correlated with higher general health scores. Therefore, more negative environments with respect to work-related bullying were associated with worse overall health. And d. the three aforementioned variables accounted for 26 % of the variance in general health scores.

## Discussion

A considerable number of studies have revealed that bullying has higher prevalence in nursing body than in other professionals [27–32].

The phenomenon of bullying, which is an extremely serious problem that affects nurses [12], can have severely negative impacts not only on satisfactory patient care but also nurses’ safety and well-being, irrespective of nurses’ ages or years of previous experience [33]. The majority of incidents of physical violence against nurses involve patients or relatives, whereas incidents of non-physical violence against nurses mainly involve other hospital staff members [34].

Bullying has physical and psychological effects on both victims and patient care organisations [35]. It can lead to depression, poor staff motivation and concentration, decreased productivity, a decreased commitment to work and poor relationships with patients, managers and colleagues [36]. Bullying can also result in occupational burnout, a lack of job satisfaction and health risks [37, 38]. On a personal level, the effects of bullying behaviours that nurses experience include helplessness, dejection, emotional pain, low self-esteem, anxiety, depression and feelings of isolation [39]. Bullying victims may also experience feelings of guilt, fear, panic and insecurity; in many cases, they exhibit psychosomatic symptoms such as depression, tearfulness, chronic post-traumatic stress disorder, nervous disorders, increased blood pressure, headaches, perspiration, nausea, vomiting, food intake disorders, sleep disorders and/or recurring nightmares [11, 40–43]. In many cases, acts of bullying can cause victims to not only evince aggressive behaviour and irritability but also resort to alcohol abuse, tranquilliser use and smoking [44].

This study’s finding suggests that bullying behaviours in the workplace have a considerable impact on nursing staff and that to varying degrees, bullying affects one-third of nursing staff. The most prevalent negative behaviours were related to work itself (unmanageable workloads, being assigned tasks below one’s level of competence) and being subjected to anger expressed by third parties.

Statistical analyses revealed that relative to other respondents, respondents who received support from their families and friends enjoyed better overall health but respondents who perceived their work environments more negatively with respect to work-related bullying suffered from worse overall health. These findings demonstrate the adverse effects that workplace bullying can have on the general health of nursing staff and the value of support systems such as family and friends.

The study findings offer valuable insights into human resource management in health services. Diagnosing the most frequent negative behaviours that unfavourably impact nurses’ health and thereby reduce the quality of the health services that nurses provide is a fundamental prerequisite for strategically planning the elimination of these behaviours. The use of appropriate tools for original evaluations of situations and for assessments of necessary interventions is a prerequisite for advancing health care organisations [29, 45].

HR management plays an important role in addressing bullying. A managerial role encompasses acknowledging, understanding and handling victims’ complaints. Difficulties faced with respect to the effective accomplishment of this role are primarily associated with HR management neglecting to provide a clear definition of bullying; ideally, HR management should specify the specific behaviours and criteria that would be regarded as bullying in a particular organisational environment [46].

### Limitations of the study

Because this investigation was quantitative and cross-sectional, it outlined workplace bullying behaviours among nursing staff within a given time frame. However, these behaviours are subject to drastic changes and alterations; therefore, the accuracy of this study’s findings may be transient. The surveyed population was recruited from public hospitals at the 1st Regional Health Service Division of Attica; thus, any attempts to generalise the study results to the entire public hospital sector should be approached with caution.

## Conclusions

Based on the findings of this study, the nursing staff of Greek hospitals are highly exposed to potential bullying. Moreover, this investigation confirmed the intrinsic nature of negative emotions resulting from bullying and revealed the value of support systems in managing the negative impact of bullying. The above information should be used to direct bullying management policies, which should be regularly reviewed using appropriate tools. Bullying is unacceptable in any context, least of all among health care professionals. It is the responsibility of all ministries of health to put procedures in place as far as possible to eradicate bullying from workplace.
